# Science Speed Dating to Spur Inter-Institutional Collaborative Research

**DOI:** 10.3390/ijerph22060919

**Published:** 2025-06-10

**Authors:** Sandra P. Chang, Kathryn L. Braun, Richard Yanagihara, Hendrik De Heer, Yan Yan Wu, Zhenbang Chen, Marc B. Cox, Stacey L. Gorniak, Georges Haddad, Christine F. Hohmann, Eun-Sook Lee, Jonathan K. Stiles, Nicolette I. Teufel-Shone, Vivek R. Nerurkar

**Affiliations:** 1 Department of Tropical Medicine, Medical Microbiology and Pharmacology, John A Burns School of Medicine, University of Hawaii at Manoa, Honolulu, HI 96813, USA; nerurkar@hawaii.edu; 2 Thompson School of Social Work & Public Health, Department of Publc Heath Studies, University of Hawai‘i at Mānoa, Honolulu, HI 96822, USA; kbraun@hawaii.edu (K.L.B.); yywu@hawaii.edu (Y.Y.W.); 3 Department of Pediatrics, John A. Burns School of Medicine, University of Hawaii at Manoa, Honolulu, HI 96826, USA; ryanagih@hawaii.edu; 4 Department of Health Sciences, Northern Arizona University, Flagstaff, AZ 86011, USA; dirk.deheer@nau.edu; 5 Department of Biochemistry, Cancer Biology, Neuroscience and Pharmacology, Meharry Medical College, Nashville, TN 37208, USA; zchen@mmc.edu; 6 Department of Biological Sciences and Border Biomedical Research Center, University of Texas at El Paso, El Paso, TX 79902, USA; mbcox@utep.edu; 7 Center for Neuromotor and Biomechanics Research, Department of Health and Human Performance, University of Houston, Houston, TX 77204, USA; sgorniak@central.uh.edu; 8 Department of Physiology and Biophysics, College of Medicine, Howard University, Washington, DC 20059, USA; ghaddad@howard.edu; 9 Department of Biology, School of Computer, Mathematical, and Natural Sciences, Morgan State University, Baltimore, MD 21251, USA; christine.hohmann@morgan.edu; 10 Department of Pharmaceutical Sciences, College of Pharmacy, Florida A&M University, Tallahassee, FL 32301, USA; eunsook.lee@famu.edu; 11 Department of Medicine, Microbiology, Biochemistry and Immunology, Clinical Research Center, Morehouse School of Medicine, Atlanta, GA 30310, USA; jstiles@msm.edu; 12 Center for Community Health and Engaged Research and Department of Health Sciences, Northern Arizona University, Flagstaff, AZ 86011, USA; nicky.teufel@nau.edu

**Keywords:** RCMI, workforce development, collaboration, networking

## Abstract

A principal strategic goal of the RCMI Coordinating Center (RCMI-CC) is to improve the health of minority populations and to reduce ethnic and geographic disparities in health by coordinating the development and facilitating the implementation of clinical research across the RCMI Consortium. To more effectively spur inter-institutional collaborative research, the RCMI-CC supports a Clinical Research Pilot Projects Program for hypothesis-driven clinical research projects proposed by postdoctoral fellows, early-career faculty and/or early-stage investigators from two or more RCMI U54 Centers. The purpose of this brief report is to summarize the Science Speed Dating sessions to facilitate cross-site collaboration at the RCMI Investigator Development Core (IDC) Workshop, held in conjunction with the 2024 RCMI Consortium National Conference. RCMI investigators and IDC Directors from 20 RCMI U54 Centers participated in two rounds of highly interactive small-group presentations of research ideas and resource needs in search of new collaborative and mentoring partnerships. Workshop participants expressed a high level of satisfaction with the speed-networking format and strongly agreed that the workshop was beneficial to their professional-development goals.

## 1. Introduction

Developing a well-trained biomedical workforce to conduct basic, behavioral, clinical, and translational research is central to the mission of the National Institutes of Health, namely, to enhance health and well-being, lengthen life, and reduce illness and disability [1]. The National Institute on Minority Health and Health Disparities (NIMHD) supports this goal through the Research Centers in Minority Institutions (RCMI) Program, which comprises the RCMI Specialized Centers (RCMI U54 Centers), the RCMI Coordinating Center (RCMI-CC), and the Clinical Research Education and Career Development (CRECD) program [2].

The RCMI Program was enacted by the US Congress in 1985 to “establish research centers in those predominantly minority institutions which offer doctoral degrees in the health professions, or the sciences related to health” [3,4]. Administered initially by the National Center for Research Resources (NCRR), the RCMI Program expanded its original focus on basic biomedical research to building research capacity for clinical research in 1995 and to developing infrastructure for community-based research in 2002. In 2007, the RCMI Translational Research Network (RTRN) was established to foster collaboration across RCMI grantee institutions with the goal of developing solutions to address health disparities in underserved communities impacted by a high disease burden. In 2011, when the NCRR was dissolved, the RCMI Program was moved to NIMHD, where it remains today.

**Figure 1:**
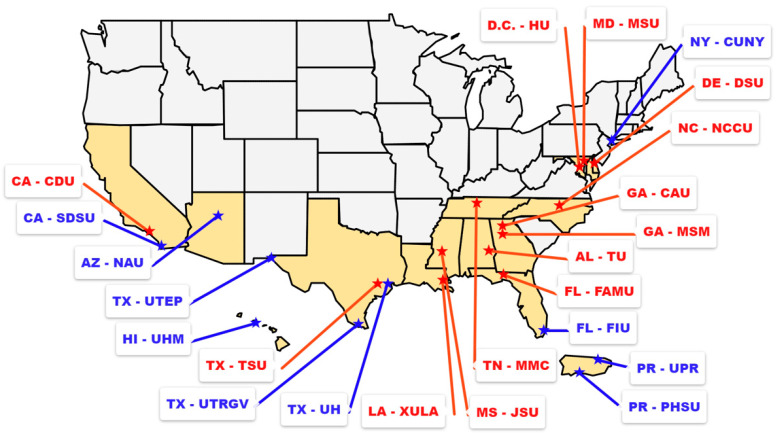
Geographic locations of the RCMI U54 Centers. Historically Black colleges and universities (HBCUs) are shown in red and non-HBCUs are shown in blue. Postal abbreviations are used for states, District of Columbia and Puerto Rico. RCMI Center abbreviations are: CAU, Clark Atlanta University; CDU, Charles R. Drew University of Medicine and Science; CUNY, City University of New York; DSU, Delaware State University; FAMU, Florida Agricultural & Mechanical University; FIU, Florida International University; HU, Howard University; JSU, Jackson State University; MMC, Meharry Medical College; MSM, Morehouse School of Medicine; MSU, Morgan State University; NCCU, North Carolina Central University; NAU, Northern Arizona University; PHSU, Ponce Health Sciences University; SDSU, San Diego State University; TSU, Texas Southern University; TU, Tuskegee University; UHM, University of Hawaii at Manoa; UH, University of Houston; UTEP, University of Texas at El Paso; UTRGV, University of Texas Rio Grande Valley; UPR, University of Puerto Rico Medical Sciences Campus; XULA, Xavier University of Louisiana.

Starting with two inaugural institutions in 1985, the RCMI Program now supports 23 RCMI U54 Centers, located in 14 states, the District of Columbia, and Puerto Rico (Figure 1). Currently, the RCMI-CC works closely with key personnel from all RCMI U54 Centers and NIMHD staff to enhance research capacity and foster faculty development at RCMI U54 Centers, to advance world-class basic biomedical, behavioral, and/or clinical research (patient-oriented and health services research), particularly on diseases and/or conditions that disproportionately impact racial and ethnic minority populations and other underrepresented populations experiencing health disparities.

Each year, the RCMI Consortium National Conference brings together scientists from all of the RCMI U54 Centers to share ideas, exchange information, and discuss opportunities for collaboration and strategies to solve shared challenges in scientific workforce development. The goal of the 2024 RCMI Consortium National Conference, convened on April 29 to May 1, was to discuss strategies for (1) project administration and coordination among sites; (2) research resources to support scientific and multi-site projects; (3) professional development and mentoring of early-stage investigators; and (4) community engagement. The Conference featured keynote speakers who highlighted the RCMI contributions to science, as well as plenary sessions by invited speakers, oral presentations by each of the RCMI U54 Centers, and poster presentations by RCMI investigators. Workshops on strategies and collaborative approaches between RCMI grantee institutions were organized by consortiums of each core. For example, the Directors of each RCMI Investigator Development Core joined together to develop a workshop for the 2024 Conference. Similarly, the Research Capacity Core (RCC) Consortium, and the Community Engagement Core (CEC) Consortium organized workshops of interest to their constituents.

The IDC Consortium Workshop, which is organized by the RCMI-CC in close partnership with the IDC Directors, has been a central feature of the annual RCMI Consortium National Conference. The format of the workshop has varied from year to year, and it has attracted participation by IDC Directors and early-stage investigators from across the RCMI U54 Centers. The 2019 IDC Workshop consisted of problem-solving group discussions that included a general information-sharing session, a discussion of best practices for mentoring, a review of professional development and pilot projects programs, and the development of a workshop manuscript [5]. The 2021 IDC Workshop featured presentations by several IDC Directors on best practices and discussions of early-stage investigator research funding opportunities led by the NIH Division of AIDS and the National Institute on Drug Abuse. The 2022 IDC Workshop was held virtually, with presentations by senior scientists on preparation of the Specific Aims page for NIH grant applications and by NIH Center for Scientific Review staff on the NIH grants-review process. The 2023 IDC Workshop focused on preparing early-stage investigators to apply for the newly established RCMI-CC Clinical Research Pilot Projects Program.

These workshops were well attended, and surveys indicated a high level of satisfaction. The purpose of this brief report is to describe and to provide evaluation findings for the 2024 IDC Consortium Workshop.

## 2. Materials and Methods

### 2.1. Workshop Format

The 2024 IDC Consortium Workshop was developed as an interactive session facilitated by one of the RCMI-CC Multiple Principal Investigators (MPI), Dr. Vivek Nerurkar, and moderated by IDC Directors from nine RCMI U54 Centers. The Workshop objectives were to provide RCMI investigators with an opportunity to explore new collaborative and mentoring partnerships, and to gain insights into research across the RCMI Consortium.

The topic and format for the IDC workshop was selected by the IDC Directors from the RCMI U54 centers. A list of potential topics was generated at an IDC Directors meeting, and a survey listing these topics was sent to IDC Directors of all RCMI grantee institutions (Table 1) for their rating. Note that the 23rd RCMI institution, CUNY, received their award after the conference was held. Topics were rated using a score of 1 for the highest priority topics and 5 for the lowest priority topics. Sixteen of the 22 institutions (73%) submitted responses, with the “speed-dating” topic receiving the highest priority score.

The “Science Speed-Dating”, or speed networking, format was designed to maximize interactions among participants during the allotted 90-min period. The aim of this approach was to help investigators build research networks and to learn more about the resources available across the RCMI Consortium. Because the RCMI-CC was offering funding to support cross-institution clinical research projects, requiring MPI from two or more RCMI Centers, the IDC Directors especially wanted to help investigators identify potential research partners at other RCMI Centers. The overall consensus was that a speed-dating format would allow participants to interact face-to-face to share information on research topics, funding mechanisms, and individual professional trajectories.

It was decided that speed-dating groups be organized around health-related or disease-focused topics, including Cancer, Environmental Health, HIV/AIDS, Infectious Diseases and Immunology, Metabolic Syndromes, Neurological Disorders and Mental Health, and Women’s Health. Prior to the workshop, IDC Directors with expertise in these areas volunteered to serve as group moderators. It was anticipated that each speed-dating group would host up to seven investigators.

Well before the workshop, IDC Directors were provided with guidelines for the speed-dating presentations and instructed to share these with their investigators to prepare for the workshop. Workshop attendees were instructed to prepare a 3-min talk, consisting of (1) a 1-min elevator pitch about themselves; (2) a 1-min description of their current research activities; and (3) a 1-min description of their research needs, such as a description of clinical samples required for their research, desired collaborations, desired mentors, and core support needs. Names and contact information were also requested for potential workshop participants, along with their research area, and whether they were basic biomedical, behavioral, community, or clinical researchers.

The 2024 IDC Consortium Workshop started with an overview presentation of the workshop objectives and its format. It was explained that two speed-dating rounds would be included within the 90-min period, allowing investigators to attend two different research-topic groups. Guidelines for the informal, oral presentations by each member of the discussion groups were included in the introduction.

Each discussion group was facilitated by a moderator who was either an IDC Director or a representative of the RCMI-CC IDC. Within the group, each participant was given 3–5 min to introduce themselves, their research interests, experimental plans, and scientific needs in a group discussion format. Most participants were new faculty members and postdoctoral fellows early in the development of their research careers and were eager to develop partnerships and to expand their networks. Following each participant’s presentation, group members were encouraged to ask questions and explore potential collaborations and areas of common interest. Those with similar interests exchanged contact information and were encouraged to meet outside of the workshop.

After about 35 min, investigators rotated to another group or remained in their original group. The workshop ended with a wrap up by the facilitator. After the workshop concluded, the IDC Directors huddled for a verbal evaluation of the workshop. Workshop moderators recorded the names, institutions, and research interests of each of the participants. Additional notes about the workshop participants’ research needs were recorded by the moderators to allow the IDC Consortium to identify potential inter-institutional collaborators for RCMI investigators.

Following the conference, workshop organizers compiled participant lists, contact information, and workshop notes from the moderators. This information was shared with all IDC Directors, who were advised to transmit this information to their investigators for follow-up on potential collaborations and networking opportunities.

### 2.2. Evaluation Questionnaire

The IDC Consortium Workshop was evaluated using a five-item online questionnaire using a QR code provided during the workshop and completed after the session. The first question asked participants to rate their overall satisfaction with the workshop on a 5-point Likert scale, from 1 = not at all satisfied to 5 = very satisfied. Then, participants were asked to specify their level of agreement (from 1 = strongly disagree to 5 = strongly agree) with four statements: (1) the workshop format and content were relevant to my professional development goals; (2) the workshop provided information about research cores and other network resources; (3) the workshop allowed me to gain new insights into research across the network; and (4) the workshop allowed me to explore new collaborative and mentoring partnerships. Data were analyzed using R statistical software (version 4.3.3, R Foundation for Statistical Computing, Vienna, Austria).

## 3. Results

### 3.1. Participants

Overall, 63 individuals from 20 RCMI U54 Centers, including 11 historically Black colleges and universities (HBCUs), participated in the IDC Workshop. This corresponded to 43 RCMI investigators and 20 IDC Directors. The numbers of participants in both speed-dating rounds for each of the various research groups were Cancer (*n* = 17), Neurological Disorders and Mental Health (*n* = 13), Women’s Health (*n* = 12), Infectious Diseases and Immunology (*n* = 9), HIV/AIDS (*n* = 8), Metabolic Syndromes (*n* = 8), and Environmental Health (*n* = 5). Of the RCMI investigators participating in the workshop, 24 of 43 (56%) completed the workshop evaluation questionnaire. This included six postdoctoral fellows, seven assistant professors, six associate or full professors, and five of unspecified rank. Only three of 20 IDC Directors completed the survey and were included in the associate or full professor group. The low response rate of IDC Directors was likely due to their reluctance to bias the survey results, since many of them participated in planning the workshop.

### 3.2. Evaluation 

Generally, participants expressed high satisfaction with the workshop (mean = 4.3, maximum score of 5.0). Participants indicated a high level of agreement that the workshop was relevant to their professional development goals (mean = 4.3), allowed them to gain new insights into research across the network (mean = 4.1), and allowed them to explore new collaborative and mentoring partnerships (mean = 4.2). The item related to gaining information about research cores and other network resources scored a bit lower (mean = 3.9). In the informal evaluation session with IDC Directors, the vast majority felt that the workshop went well, and several provided examples of promising linkages between participants in their speed-dating groups. As shown in Table 2, the mean scores from postdoctoral fellows were about 0.3 to 0.5 lower than that of the assistant professors, and the means from associate or full professor were about 0.1 to 0.2 lower than that of assistant professors as well. However, these differences were not statistically significant at the 5% level.

### 3.3. Outcomes

One of the objectives of the IDC Consortium Workshop was to enhance collaborative, inter-institutional MPI applications for the RCMI-CC Clinical Research Pilot Projects Program. Table 3 summarizes the profiles of applicants to the pilot project program. Twenty-four applications to this program were received over three cycles, from 2023 to 2024. These applications represented 40 MPI from 16 RCMI grantee institutions. Of these MPI applicants, 17 were from HBCUs and 23 were from non-HBCU RCMI U54 Centers. The number of female applicants exceeded male applicants in both submitted and funded applications. Thirteen of the 24 applications (54%) were funded, representing seven MPI from HBCUs and 19 MPI from non-HBCUs. These pilot projects are ongoing, and the long-term success of these projects in generating collaborative manuscripts and new grant applications is being tracked.

## 4. Discussion

The 2024 IDC Consortium Workshop was well-attended with participating RCMI investigators and IDC Directors representing 20 of 22 (91%) RCMI U54 Centers participating in the conference. The “science speed-dating” format was highly interactive and enabled investigators to identify potential collaborators and IDC Directors to increase their awareness of the research expertise and interests at other institutions within the RCMI Consortium.

A high level of overall satisfaction with the IDC Workshop was expressed by participants at all academic ranks, from postdoctoral fellows to full professors and IDC Directors. Participants strongly agreed that the workshop was relevant to their professional-development goals. A major objective of the workshop was to identify potential collaborations for early-stage investigators planning to prepare an application for the RCMI-CC Clinical Research Pilot Projects Program that required the formation of partnerships of MPI from two or more RCMI U54 Centers. Therefore, it was important that participants strongly agreed that the IDC Workshop allowed them to gain new insights into research across the RCMI Consortium and to explore new collaborative and mentoring partnerships. Long-term tracking of investigators participating in the IDC Workshop is needed to provide insights into whether it contributed to the establishment of inter-instititional research collaborations, publication of co-authored manuscripts, and submission of collaborative MPI grant applications.

Science Speed-Dating has been previously used to stimulate the formation of new interdisciplinary research collaborations. In one instance, speed-networking sessions were offered in the context of scientific retreats where presenters met with attendees to exchange ideas and discuss collaborations [6]. In another example, a community-based participatory research speed-dating (CBPR-SD) technique was used to promote and establish relationships between researchers and community stakeholders prior to the development and execution of CBPR projects [7]. In both examples, as well as in the current case, these speed-dating sessions were intended to initiate a collaboration that subsequently would allow the team to compete for interdisciplinary, collaborative pilot project research funding.

The Science Speed Dating approach is similar to the “Un-Meeting” conference format developed by the NIH Clinical and Translational Science Awards (CTSA) program to foster new collaborations and innovative thinking at conferences through networking and engaged discussions across diverse groups [8]. These interactive, non-traditional “un-meetings” consist of 4-min lightning talks and generation of topic ideas for follow-up breakout sessions. Survey results of this meeting format indicated participation by a broad set of attendees, a high level of networking during the meeting, and the potential to advance team and translational science.

Team science, defined by the National Research Council as “scientific collaboration by more than one individual in an interdependent fashion, including research conducted by small teams and larger groups” [9], has been associated with increased productivity and collaboration [10]. Team-science pilot projects programs have been successful at individual RCMI U54 Centers [11], and hands-on training in strategic team science was offered at a previous RCMI Consortium National Conference [12]. Bringing investigators together from different disciplines and institutions to work as teams at an early-career stage is important for scientific discovery and productivity. Prior research on scientific networks has shown that teams with mixed institutional associations and high disciplinary diversity were more productive and central to the overall network [13]. A recent analysis of a cross-institutional network of three institutions conducting health disparities research over a 10-year period showed growth of the network by 25-fold, with the most productive cluster involving the most different disciplines and participants from all three institutions [14].

In addition to cross-disciplinary membership, teams that also include investigators representing various demographics and settings, outperform homogeneous investigative teams [15,16,17,18]. Diversity in researcher demographics can increase our ability to identify and address health issues that tend to plague some segments of the population more than others. For example, engaging a Filipino researcher in studies to address Filipino health issues will likely improve scientific hypotheses, intervention designs, recruitment strategies, retention in research, and applicability of findings to the Filipino population in the U.S. The same can be said of engaging female investigators in research addressing women’s health and non-straight investigators in research focusing on issues affecting non-straight Americans. Diversity in investigator setting—academia, health care facility, or community—will allow a research team to design and conduct research that answers real-world clinical and community questions and produce findings that lead to tailored interventions that reflect a facility’s or population’s culture and context [19,20,21]. Thus, NIH has supported the professional and demographic diversification of the clinical and translational research workforce to foster scientific innovation, increase the quality of research, advance participation of diverse populations in research, and ultimately lead to acceptable, accessible, and successful interventions [22].

The IDC Consortium Workshop was one component of the RCMI-CC’s outreach efforts to increase the awareness of shared research interests of early-stage investigators at different RCMI U54 Centers and to promote the formation of collaborative teams to apply for pilot project funding. Other outreach activities included the 2023 RCMI IDC Workshop which focused on increasing overall awareness of this new funding opportunity. In addition, the RCMI-CC arranged for early-stage investigators to present short talks on their research topics and interests in a consortium-wide webinar series, with the objective of identifying collaborators for the pilot projects program.

Limitations of this brief report include the descriptive nature of the workshop using a speed-dating approach to develop cross-institution research teams and the pre-experimental nature of our evaluation. Unfortunately, the conference venue only allowed us to offer the workshop once and to accept all interested attendees, and there was no option for randomization or creation of a control group. Also, we were limited to having small-group interactive presentations, instead of one-on-one focused exchanges. Data on long-term outcomes are still being tracked, so we can only speculate about the benefits of this workshop on sustained collaborative or mentoring partnerships.

Nevertheless, short-term outcomes are gratifying. As a result of these efforts, the RCMI-CC Clinical Research Pilot Projects Program received a total of 24 pilot project applications from 40 MPI, representing 16 of the 23 (70%) RCMI U54 Centers. The applicant MPIs were diverse in gender and represented both HBCU and non-HBCU institutions. Of these, 13 pilot projects, led by 26 MPI, were selected for funding and are currently in progress.

## 5. Conclusions

The RCMI Consortium National Conference serves as a forum for the RCMI Specialized Centers to share administrative strategies, professional-development issues, community-engagement approaches, and research accomplishments. The IDC Consortium Workshop planned by the RCMI-CC and RCMI U54 IDC Directors is a central feature of the conference. In 2024, the IDC Consortium Workshop implemented a “science speed-dating” activity to foster new collaborative and mentoring partnerships and provide participants with insights into research across the RCMI Consortium. This workshop was well-attended, and survey evaluation results indicated high satisfaction and fulfillment of the workshop objectives. This workshop, along with other RCMI-CC outreach activities, resulted in the development of new inter-institutional collaborations and increased MPI-led pilot project applications.

## Figures and Tables

**Table 1:** Suggested IDC workshop topics and priority score.

Suggested Workshop Topic	Avg. Priority Score *
“Speed-dating”—an opportunity for early-stage and established investigators to meet and to discuss their research to foster collaborations and mentoring	1.9
Presentations of NIH funding opportunities by NIH program officials (K including K99/R00; R15/R16; High Risk-High Reward DP1, DP2, DP5)	2.0
Best practices for Individual Development Plans and Mentor-Mentee agreements	2.4
Best practices for the responsible use of artificial intelligence (AI) and machine learning in biomedical research proposals	3.0
Best practices for enhancing mentoring skills; mentoring the mentor	3.2
Best practices for overcoming barriers to professional development of postdoctoral fellows and early-stage investigators	3.4
Best practices for facilitating transdisciplinary research and team science (integration of basic, clinical, behavioral, community, and population science)	3.6
Understanding rigor and reproducibility in biomedical research proposals	3.8

* Scoring scale: 1 = Highest Priority to 5 = Lowest Priority.

**Table 2:** Mean scores and standard deviations (SD) of the questionnaires for the total sample and by participants’ rank *.

Total Sample *n* = 24	Postdoctoral Fellow *n* = 6 (25%)	Assistant Professor *n* = 7 (29%)	Associate or Full Professor *n* = 6 (25%)	Other *n* = 5 (21%)
Mean (SD)	Mean (SD)	Mean (SD)	Mean (SD)	Mean (SD)
How satisfied were you with this workshop? (Range 3 to 5)				
4.3 (0.9)	4.0 (0.9)	4.4 (0.8)	4.2 (1.0)	4.6 (0.9)
Q1. The workshop format and content were relevant to my professional development goals (Range 3 to 5)				
4.3 (0.8)	4.0 (0.9)	4.4 (0.8)	4.3 (0.8)	4.4 (0.5)
Q2. The workshop provided information about research cores and other network resources (Range 3 to 5)				
3.9 (1.1)	3.7 (1.4)	4.1 (0.9)	3.8 (1.2)	4.0 (1.2)
Q3. The workshop allowed me to gain new insights into research across the network (Range 3 to 5)				
4.1 (0.8)	3.8 (0.8)	4.1 (0.7)	4.2 (1.0)	4.4 (0.9)
Q4. The workshop allowed me to explore new collaborative and mentoring partnerships (Range 3 to 5)				
4.2 (0.8)	3.8 (0.8)	4.3 (0.8)	4.2 (1.0)	4.4 (0.9)

* Parametric and non-parametric one-way ANOVA tests for difference in means were used to compute *p*-values. All *p*-values for the tests of differences between postdoctoral fellows and assistant professors are greater than 0.28. All *p*-values for the tests of differences among postdoctoral fellows, assistant professors and associate or full Professors are greater than 0.58. All *p*-values for the tests of differences among the four groups are greater than 0.60.

**Table 3:** RCMI Clinical Research Pilot Projects Program, 2023–2024.

Metric	No. Submitted	No. Funded
Collaborative pilot project applications	24 *	13
MPI applicants	40	26
MPI applicants Female	28	16
MPI applicants Male	12	10
MPI applicants from HBCU	17	7
MPI applicants from non-HBCU	23	19
Participating RCMI grantee institutions	16	12

* Includes resubmissions.

## Data Availability

The original contributions presented in this study are included in the article. Further inquiries can be directed to the corresponding author.
